# Sum-of-squares of polynomials approach to nonlinear stability of fluid flows: an example of application

**DOI:** 10.1098/rspa.2015.0622

**Published:** 2015-11-08

**Authors:** D. Huang, S. Chernyshenko, P. Goulart, D. Lasagna, O. Tutty, F. Fuentes

**Affiliations:** 1 Department of Aeronautics, Imperial College London, Prince Consort Road, London SW7 2AZ, UK; 2 Department of Engineering Science, University of Oxford, Parks Road, Oxford OX1 3PJ, UK; 3 Engineering and the Environment, University of Southampton, Highfield, Southampton SO17 1BJ, UK; 4 Institute for Computational Engineering and Sciences (ICES), The University of Texas at Austin, 201 East 24th Street, Austin, TX 78712, USA

**Keywords:** flow stability, rotating Couette flow, Lyapunov function, sum-of-squares of polynomials, semi-definite programming

## Abstract

With the goal of providing the first example of application of a recently proposed method, thus demonstrating its ability to give results in principle, global stability of a version of the rotating Couette flow is examined. The flow depends on the Reynolds number and a parameter characterizing the magnitude of the Coriolis force. By converting the original Navier–Stokes equations to a finite-dimensional uncertain dynamical system using a partial Galerkin expansion, high-degree polynomial Lyapunov functionals were found by sum-of-squares of polynomials optimization. It is demonstrated that the proposed method allows obtaining the exact global stability limit for this flow in a range of values of the parameter characterizing the Coriolis force. Outside this range a lower bound for the global stability limit was obtained, which is still better than the energy stability limit. In the course of the study, several results meaningful in the context of the method used were also obtained. Overall, the results obtained demonstrate the applicability of the recently proposed approach to global stability of the fluid flows. To the best of our knowledge, it is the first case in which global stability of a fluid flow has been proved by a generic method for the value of a Reynolds number greater than that which could be achieved with the energy stability approach.

## 1. Introduction

Hydrodynamic stability is the field that investigates the transient effects of an initial perturbation of a known steady flow. The area has attracted the attention of many researchers and is closely related to the study of transition to turbulence [1,2].

Using Lyapunov stability theory, a steady flow can be proved to be stable with respect to perturbations of arbitrary amplitude by constructing a Lyapunov functional *V* [**u**], which is a positive-definite functional of the velocity perturbation **u** that decays monotonically on any non-zero solution **u**(*t*,**x**) of the Navier–Stokes equations [3].

Usually, the Lyapunov functional is chosen to be the perturbation energy *E*=∥**u**∥^2^/2, where the norm is defined as an integral of |**u**|^2^ over the flow domain. Then the problem of proving that *E* is a Lyapunov functional reduces to a tractable linear eigenvalue problem [4,5]. However, the resulting estimates of the global stability range, usually expressed by the energy stability limit Reynolds number *Re*_E_, could be very conservative in the sense that *Re*_E_ is generally far below the maximum *Re* for which the flow is globally stable.

Recently, a method was proposed by Goulart & Chernyshenko [6] for exploiting the sum-of-squares (SOS) decomposition [7,8] to construct polynomial Lyapunov functionals differing from *E*, thus extending the range of *Re* in which the flow can be proved to be globally stable. In this approach, the Navier–Stokes equations are first reduced to a finite-dimensional uncertain dynamical system, that is a system of ordinary differential equations (ODEs) with right-hand side containing terms for which only bounds, but not exact expressions, are available. For incompressible flows both the right-hand side of the ODEs and the bounds are polynomial. The corresponding Lyapunov stability condition can then be reduced to a condition of positive definiteness of other certain polynomials [6]. Noting that the condition of a polynomial being positive-definite can be replaced by a stronger, but more tractable numerically, condition of the polynomial being a SOS of other polynomials, an admissible Lyapunov functionals can be found using the polynomial SOS optimization approach [7,9].

The SOS technique has been applied in stability analysis of constrained ODEs [10], hybrid systems [8] or time-delay systems [11], but there are few results for partial differential equations. The relevant publications are [12] and [13], where SOS-based algorithmic methodologies are presented for the analysis of systems described by certain types of parabolic partial differential equations. It was shown how certain Lyapunov structures could be constructed to prove stability using transformations defined through integration by parts. It is worth noting that in both [12,13], the partial differential equations are considered directly, which is different from the construction process of a Lyapunov functional in [6].

Application of SOS of polynomials to fluid flows goes beyond nonlinear stability. After an overview of nonlinear stability applications including a short announcement of a part of the results of this work, applications for deriving rigorous bounds on time-averaged characteristics of turbulent flows and applications to flow control are discussed in [14].

While [6] provides a full theoretical description of the new approach, it was applied there only to a truncated Galerkin approximation rather than to the full Navier–Stokes equations, thus leaving open the question of whether there is at least one fluid flow for which this method will work. Such an example is given in this paper. To the best of our knowledge, this study is the first case in which global stability of a fluid flow has been proved by a generic method for the value of a Reynolds number greater than that which could be achieved with the energy stability approach.

## 2. Problem formulation

Our goal is to demonstrate that the method proposed in [6] can actually be used to prove the stability of a fluid flow for the values of the Reynolds number above the energy stability limit. This section describes briefly the method and the flow to which it is to be applied for achieving this goal.

### (a) The method

An unsteady flow of incompressible viscous fluid in a given domain with time-independent boundary conditions is considered. The perturbation velocity **u**(*t*,**x**), defined as the deviation of the instantaneous flow velocity from the steady solution $\bar{\text{u}}$, is governed by the Navier–Stokes equations with an additional linear term:

2.1 $$\frac{\partial \text{u}}{\partial t}+\text{u}\cdot \nabla \text{u}=-\nabla p+\frac{1}{Re}\nabla^{2}\text{u}+A\text{u},\;\nabla \cdot \text{u}=0.$$

The linear operator *A* depends on the flow in question. It is convenient to leave it in a compact general form here. This formulation is a little more general than in [6], where *A* had a particular form, but all the results of Goulart & Chernyshenko [6] apply with obvious minor modifications, which are made without further comments in the summary of the method given in this section. The perturbation velocity **u** is subject to homogeneous boundary conditions. The steady flow can be proved to be stable with respect to perturbations of arbitrary amplitude by constructing a Lyapunov functional *V* [**u**].

#### (i) The Lyapunov functional

For this purpose, in [6] the perturbation velocity is represented as

2.2 $$\text{u}(\text{x},t)=\sum_{i=1}^{k}a_{i}(t){\text{u}}_{i}(\text{x})+{\text{u}}_{s}(\text{x},t),$$

where the finite Galerkin basis fields **u**_*i*_, *i*=1,…,*k*, are an orthonormal set of solenoidal vector fields with the inner product 〈**w**_1_,**w**_2_〉 defined as the integral of **w**_1_⋅**w**_2_ over the flow domain V, the residual perturbation velocity **u**_*s*_ is solenoidal and orthogonal to all the **u**_*i*_, and both **u**_*i*_ and **u**_*s*_ satisfy the homogeneous boundary conditions. The Lyapunov functional is sought in the form^1^ *V* [**u**]=*V* (**a**,*q*^2^), where **a**=(*a*_1_,…,*a*_*k*_), and *q*^2^=∥**u**_*s*_∥^2^/2=〈**u**_*s*_,**u**_*s*_〉/2. For *V* [**u**] to be a Lyapunov functional, the function $V(\text{a},q^{2}):R^{k}\times R\to R$ should be positive-definite, and its value should decrease monotonically towards zero along all possible non-zero solutions of (2.1): *V* (**a**,*q*^2^)>0 and d*V*/d*t*<0, for all (**a**,*q*^2^)≠0. With the use of (2.1) and (2.2), the latter condition can be rewritten as [6]

2.3 $$\frac{dV}{dt}=\frac{\partial V}{\partial \text{a}}\cdot \text{f}(\text{a})+\frac{\partial V}{\partial (q^{2})}(\Gamma ({\text{u}}_{s})+\chi ({\text{u}}_{s},\text{a}))+\left(\frac{\partial V}{\partial \text{a}}-\frac{\partial V}{\partial (q^{2})}\text{a}\right)\cdot \Theta ({\text{u}}_{s},\text{a})<0,$$

where the components of the vector **f** are

2.4 $$f_{i}(\text{a})=L_{ij}a_{j}+N_{ijk}a_{j}a_{k},\;L_{ij}=\frac{1}{Re}\langle {\text{u}}_{i},\nabla^{2}{\text{u}}_{j}\rangle +\langle {\text{u}}_{i},A{\text{u}}_{j}\rangle \;\mathrm{and}\;N_{ijk}=-\langle {\text{u}}_{i},{\text{u}}_{j}\cdot \nabla {\text{u}}_{k}\rangle ,$$

the scalar functionals *Γ* and *χ* are^2^

2.5 $$\Gamma ({\text{u}}_{s})=\frac{1}{Re}\langle {\text{u}}_{s},\nabla^{2}{\text{u}}_{s}\rangle -\langle {\text{u}}_{s},{\text{u}}_{s}\cdot \nabla \bar{\text{u}}+\bar{\text{u}}\cdot \nabla {\text{u}}_{s}\rangle$$

and

2.6 $$\chi ({\text{u}}_{s},\text{a})=\langle {\text{u}}_{s},{\text{g}}_{j}\rangle a_{j},\;{\text{g}}_{j}=\frac{2}{Re}\nabla^{2}{\text{u}}_{j}-({\text{u}}_{j}\cdot \nabla \bar{\text{u}}+\nabla \bar{\text{u}}\cdot {\text{u}}_{j}),$$

where $\bar{\text{u}}$ is the steady flow the stability of which is studied, and the vector-valued functional **Θ**(**u**_*s*_,**a**)=**Θ**_*a*_(**u**_*s*_)+**Θ**_*b*_(**u**_*s*_,**a**)+**Θ**_*c*_(**u**_*s*_) has the components

2.7 $$\Theta_{ai}({\text{u}}_{s})=\langle {\text{u}}_{s},{\text{h}}_{i}\rangle ,\;\Theta_{bi}({\text{u}}_{s},\text{a})=\langle {\text{u}}_{s},{\text{h}}_{ij}\rangle a_{j}\;\mathrm{and}\;\Theta_{ci}({\text{u}}_{s})=\langle {\text{u}}_{s},{\text{u}}_{s}\cdot \nabla {\text{u}}_{i}\rangle ,$$

where ${\text{h}}_{i}=(1/Re)\nabla^{2}{\text{u}}_{i}+\bar{\text{u}}\cdot \nabla {\text{u}}_{i}-\nabla \bar{\text{u}}\cdot {\text{u}}_{i}$ and **h**_*ij*_=**u**_*j*_⋅∇**u**_*i*_−∇**u**_*j*_⋅**u**_*i*_. The notation used can be clarified by the Einstein equivalent of the formula for **h**_*i*_: $h_{i}^{m}=(1/Re)\nabla^{2}u_{i}^{m}+{\bar{u}}^{k}(\partial u_{i}^{m}/\partial x^{k})-(\partial {\bar{u}}^{k}/\partial x^{m})u_{i}^{k}$, where $h_{i}^{m},$ $u_{i}^{m}$ and *x*^*m*^ are the *m*th components of the vectors **h**_*i*_, **u**_*i*_ and **x**, respectively.

Constructing *V* satisfying (2.3) and *V* >0 for all (**a**,*q*^2^)≠0 would prove the global stability of the flow under consideration. However, (2.3) involves **u**_*s*_ while *V* is a function of **a** and *q*^2^ only. Hence the next step is required [6].

#### (ii) The bounds

The terms in (2.3) dependent on **u**_*s*_ can be bounded by functions of **a** and *q*^2^. Note that *χ*, *Θ*_*ai*_, and *Θ*_*bi*_ are linear functionals of **u**_*s*_, while *Γ* and *Θ*_*ci*_ are quadratic functionals of **u**_*s*_.

For *Θ*_*ai*_ defined by (2.7) the Cauchy–Schwarz inequality gives $|\Theta_{ai}({\text{u}}_{s})|\leq ∥{\text{u}}_{s}∥∥{\text{h}}_{i}∥=\sqrt{1/2}|q|∥{\text{h}}_{i}∥.$ Obtaining the tight bound requires projecting **h**_*i*_ onto the solenoidal subspace and a small modification to account for the boundary conditions [6]. Other linear functionals can be bounded similarly.

The linear functional *χ* is a special case. If the basis **u**_*j*_ is chosen to consist of the eigenfunctions of the classical energy stability problem [15] for $\bar{\text{u}},$ then *χ*≡0 [6]. This can become obvious if one notices the similarity between the energy stability operator [15] and (2.6). We will use such a basis in this study. Accordingly, even though we refer to the following formulae as obtained in [6], in fact they are simplified versions that we derived with an additional assumption *χ*=0. In most cases, this can be done by simply omitting some terms. The readers wishing to use the method with the basis **u**_*i*_ in which *χ*≠0 should refer to [6] rather than to the formulae below.

The bounds on the quadratic functionals can be obtained by maximizing the functionals subject to a constraint *q*^2^=1. This reduces to a linear eigenvalue problem in a fairly standard way. The resulting bound for *Γ* has the form [6]

2.8 $$\Gamma ({\text{u}}_{s})\leq \kappa_{s}q^{2}.$$

Note the link to the energy stability problem following from the form of (2.5): finding *κ*_*s*_ reduces to the energy stability problem with an additional constraint 〈**u**_*s*_,**u**_*i*_〉=0 ∀ *i*. When the linear eigenvalue problem has a discrete set of eigenvalues, only a finite number of them can be positive [15] and, hence, *κ*_*s*_ can be made negative by selecting a suitable basis **u**_*i*_.

Rather than using the generic approach to bounding *Θ*_*ci*_ as proposed in [6], in appendix A we derive an explicit tight bound for it.

Putting together all the above gives the following set of bounds

2.9 $$\chi =0,\;\Gamma ({\text{u}}_{s})\leq \kappa_{s}q^{2},\;\kappa_{s}<0,\;|\Theta ({\text{u}}_{s},\text{a}){|}^{2}\leq p(\text{a},q^{2}),$$

where the particular values of *κ*_*s*_ and the coefficients of the quadratic polynomial *p*(**a**,*q*^2^) depend on the particular flow in question and on the selection of the particular eigenfunctions of the corresponding energy stability problem to be used as the finite basis **u**_*i*_.

This allows formulating the stability analysis problem as a SOS optimization problem.

#### (iii) Reduction to a polynomial sum-of-squares optimization

Since the term *Γ*(**u**_*s*_) in (2.3) is upper-bounded by a negative-definite function *κ*_*s*_*q*^2^ but is not lower-bounded, one has to impose an additional requirement that the candidate function *V* satisfy

2.10 $$\frac{\partial V}{\partial (q^{2})}\geq 0,\;\forall (\text{a},q^{2})\neq 0.$$

The condition (2.10) ensures that the term (∂*V* /∂(*q*^2^))*Γ*(**u**_*s*_) makes a negative contribution to the left-hand side of the Lyapunov condition (2.3). Combining the condition (2.10) with the bounds (2.9) gives that (2.3) holds if

2.11 $$\frac{\partial V}{\partial \text{a}}\cdot \text{f}+\frac{\partial V}{\partial (q^{2})}\kappa_{s}q^{2}+\left|\frac{\partial V}{\partial \text{a}}-\frac{\partial V}{\partial (q^{2})}\text{a}\right|p^{1/2}(\text{a},q)<0,\;\forall \;(\text{a},q^{2})\neq 0.$$

The condition (2.11) is difficult to use because both *V* and *p* enter it nonlinearly. This can be circumvented via introduction of additional variables. As shown in [6], (2.11) is equivalent to

2.12 $${\text{z}}^{T}H(\text{a},q^{2})\text{z}>0,\;\forall \;\text{z}\neq 0,\;\forall \;(\text{a},q)\neq 0,$$

where

2.13 $$H(\text{a},q^{2})=\left(\begin{array}{cc} -s_{1}(\text{a},q^{2})p(\text{a},q^{2}) & {\text{s}}_{2}(\text{a},q^{2})\cdot p(\text{a},q^{2})\\ {\text{s}}_{2}^{T}(\text{a},q^{2})\cdot p(\text{a},q^{2}) & -s_{1}(\text{a},q^{2})I \end{array}\right)$$

and

$$s_{1}(\text{a},q^{2})=\frac{\partial V}{\partial \text{a}}\text{f}+\frac{\partial V}{\partial (q^{2})}\cdot \kappa_{s}q^{2},\;{\text{s}}_{2}(\text{a},q^{2})=\frac{\partial V}{\partial \text{a}}-\frac{\partial V}{\partial (q^{2})}{\text{a}}^{T}.$$

In summary, if one is able to construct a function *V* simultaneously satisfying the three conditions *V* >0, (2.10) and (2.12), for all (**a**,*q*^2^≠0) then the flow in question is globally stable.

If *V* is sought for in the form of a polynomial then checking each of these three conditions amounts to checking the global non-negativity of a polynomial function, which is known to be NP-hard; see [9]. However, a sufficient, and numerically tractable, condition for global non-negativity of a polynomial is that it can be written as a SOS of other polynomials. Accordingly, a sufficient condition for joint satisfaction of our three conditions is that

2.14 $$V(\text{a},q^{2})-\ell_{1}(\text{a},q^{2})\in \Sigma_{k+1},\;\frac{\partial V}{\partial (q^{2})}\in \Sigma_{k+1}\;\text{and}\;\text{z}H(\text{a},q)\text{z}-\ell_{3}(\text{a},q^{2},\text{z})\in \Sigma_{2k+2},$$

where *Σ*_*l*_ represents the set of all SOS polynomials in $R^{l}$, and the positive-definite polynomial functions $\ell_{i}(\text{c})=\sum_{j}\epsilon_{ij}c_{j}^{2},\epsilon_{ij}\geq 0,\sum_{j}\epsilon_{ij}>0$ are used in place of the vector-value conditions **z**≠0 and/or (**a**,*q*^2^)≠0.

Existence of a function *V* satisfying the conditions (2.14) can be checked via the polynomial SOS approach of [7,9], which amounts to solving a convex optimization problem in the form of a large semi-definite program. For a prescribed degree of the candidate polynomial *V* , the solution to that problem either provides an explicit expression for *V* or states that such *V* does not exist. The SOS approach is well known and will not be described here.

#### (iv) Uncertain system interpretation

Substituting the partial Galerkin expansion (2.2) into the Navier–Stokes equations (2.1) and projecting onto **u**_*i*_ gives

2.15 $$\frac{d\text{a}}{dt}=\text{f}(\text{a})+\Theta ,$$

and the easily obtainable equation for the energy *q*^2^ of the residual field **u**_*s*_ is

2.16 $$\frac{dq^{2}}{dt}=-\text{a}\cdot \Theta +\Gamma +\chi ,$$

where **Θ**, *Γ* and *χ* are defined in §2a(i) as functionals of **u**_*s*_ and functions of **a**. One can, however, allow **Θ**, *Γ* and *χ* in (2.15) and (2.16) to assume any values as far as they satisfy the set of known bounds defined in terms of **u**_*s*_ and **a**, such as (2.9). In this sense, ((2.9), (2.15), (2.16)) is an uncertain dynamical system. The solution of this system is therefore not unique. However, if all the solutions of ((2.9), (2.15), (2.16)) tend to zero as time tends to infinity, then the solution of the Navier–Stokes system also tends to zero. The stability conditions described above are in fact the stability condition for this uncertain system. This turns out to be quite useful in understanding and interpretation of the further results.

Note that *V* (**a**,*q*^2^) is a Lyapunov function for the uncertain system and at the same time a Lyapunov functional for the full Navier–Stokes equations.

If one takes *V* =∥**a**∥^2^/2+*q*^2^ then (2.11) reduces to the classic energy stability problem. Hence, this approach [6] is guaranteed to give at least as good results as the energy stability approach. In order to demonstrate that it is capable of providing a better result, it has to be applied to a particular flow.

### (b) Double-periodic rotating Couette flow

Given the novelty and complexity, and the relatively demanding computational requirements for solving the semi-definite programming problems stemming from polynomial SOS optimization, for the first application of the stability analysis method of Goulart & Chernyshenko [6], it is desirable to select as simple a flow as possible. The particular flow we select is a version of the famous rotating Couette flow between two co-axial cylinders.

#### (i) Governing equations

The gap between the cylinders is assumed to be much smaller than the cylinder radius. A local Cartesian coordinate system **x**=(*x*,*y*,*z*) is oriented such that the axis of rotation is parallel to the *z*-axis, while the circumferential direction corresponds to the *x*-axis. Only flows independent of *x* are considered. The flow velocity is represented as (*y*+*u*,*v*,*w*), so that **u**=(*u*,*v*,*w*) is the velocity perturbation and $\bar{\text{u}}=(y,0,0)$ is the equilibrium flow. Under these assumptions, the governing equations are [16,17]

2.17*a* $$\begin{array}{rl} \frac{\partial u}{\partial t}+v\frac{\partial u}{\partial y}+w\frac{\partial u}{\partial z}+v & =\Omega v+\frac{1}{Re}\left(\frac{\partial^{2}u}{\partial y^{2}}+\frac{\partial^{2}u}{\partial z^{2}}\right), \end{array}$$

2.17*b* $$\begin{array}{rl} \frac{\partial v}{\partial t}+v\frac{\partial v}{\partial y}+w\frac{\partial v}{\partial z} & =-\Omega u-\frac{\partial p}{\partial y}+\frac{1}{Re}\left(\frac{\partial^{2}v}{\partial y^{2}}+\frac{\partial^{2}v}{\partial z^{2}}\right), \end{array}$$

2.17*c* $$\begin{array}{rl} \frac{\partial w}{\partial t}+v\frac{\partial w}{\partial y}+w\frac{\partial w}{\partial z} & =-\frac{\partial p}{\partial z}+\frac{1}{Re}\left(\frac{\partial^{2}w}{\partial y^{2}}+\frac{\partial^{2}w}{\partial z^{2}}\right) \end{array}$$

2.17*d* $$\begin{array}{rl} \;\text{and}\;\frac{\partial v}{\partial y}+\frac{\partial w}{\partial z} & =0, \end{array}$$

where *Ω* is a non-dimensional parameter characterizing the Coriolis force, *Re* is the Reynolds number and *p* is pressure. More compactly, (2.17) can be written in the vector form (2.1) with

$$A=\left(\begin{array}{ccc} 0 & \Omega -1 & 0\\ -\Omega & 0 & 0\\ 0 & 0 \end{array}\right).$$

In §2a, *A* was assumed to contain only the terms depending on the base flow $\bar{\text{u}},$ while here the terms with *Ω* are present. Conveniently, their presence does not affect any of the formulae in §2a, mostly because these terms correspond to the Coriolis force and thus do not enter the energy equation.

For simplicity, the flow is assumed to be 2*π*-periodic in *y* and *z*, *u* and *v* are assumed to be odd in *y* and even in *z*, while *w* is assumed odd in *z* and even in *y*:

2.18 $$\begin{array}{rl} \text{u}(y,z) & =\text{u}(y+2\pi ,z)=\text{u}(y,z+2\pi ),\;p(y,z)=p(y+2\pi ,z)=p(y,z+2\pi ),\\ u(y,z) & =-u(-y,z)=u(y,-z),\;v(y,z)=-v(-y,z)=v(y,-z)\\ \;\mathrm{and}\;w(y,z) & =w(-y,z)=-w(y,-z). \end{array}\}$$

#### (ii) Stability properties of the flow

We first apply the well-known energy stability approach. Setting the Lyapunov functional as the perturbation energy *E*=∥**u**∥^2^/2 leads to a linear eigenvalue problem [15]. For (2.17)–(2.18), the resulting eigenfunctions **e**_*n*,*m*_(**x**), as can be verified by direct substitution into that eigenvalue problem, are:

2.19 $${\text{e}}_{n,m}(\text{x})=\left(\frac{\cos(mz)\sin(ny)}{\sqrt{2}\pi },\frac{m\cos(mz)\sin(ny)}{\sqrt{2}\pi \sqrt{m^{2}+n^{2}}},-\frac{n\sin(mz)\cos(ny)}{\sqrt{2}\pi \sqrt{m^{2}+n^{2}}}\right),$$

where *n*=1,2,…,*m*=0,±1,±2,…. The corresponding eigenvalues are

2.20 $$\lambda_{n,m}(Re)=\frac{-m}{2\sqrt{m^{2}+n^{2}}}-\frac{m^{2}+n^{2}}{Re}.$$

Note that for this flow, conveniently, neither the eigenfunctions nor the eigenvectors depend on *Ω*. The eigenvalue λ_1,−1_ is positive for $Re>4\sqrt{2}$, with all other eigenvalues less than λ_1,−1_. Hence, the energy stability limit is $Re=Re_{E}=4\sqrt{2}$. One can show that the flow becomes linearly unstable for 0<*Ω*<1 and

2.21 $$Re>Re_{L}=\frac{2\sqrt{2}}{\sqrt{1-\Omega }\sqrt{\Omega }}.$$

Note that *Re*_L_=*Re*_E_ for $\Omega =\frac{1}{2}$.

For the classical case of no-slip conditions at the wall, it has been proved [18] that the linear stability and the global stability limits coincide. For the double-periodic flow considered here the same is true, too. This can be proved by the same method as in [18], which amounts to selecting the Lyapunov functional in the form

2.22 $$V=\int (\lambda u^{2}+v^{2}+w^{2})\;dy\;dz$$

and adjusting the constant λ to get as best stability limit as possible. Similar results were obtained in [19] using a more systematic approach involving convex optimization technique. These approaches cannot be applied to an arbitrary flow, unlike the method proposed in [6]. On the other hand, the form of the Lyapunov functional *V* =*V* (**a**,*q*^2^) might be too restrictive to obtain the same results as with (2.22).

Hence, for the flow we are considering both the energy stability limit and the actual global stability limit are known, giving the framework for considering the performance of the method [6].

## 3. Application of the method to the double-periodic rotating Couette flow

### (a) Selection of the Galerkin basis fields **u**_*i*_

In solving the SOS feasibility problem (2.14), the uncertain parts of the system (2.15)–(2.16) are characterized only by their bounds. Further, those bounds are linked directly to the Galerkin basis fields **u**_*i*_, *i*=1,…,*k*, as it is evident from (2.5)–(2.7). The choice of the Galerkin basis fields is therefore crucial. If *k* is too small, then the dynamic model of the system becomes over-simplified and does not adequately capture the salient features of the flow. Consequently, it might be difficult to achieve a better stability result than *Re*_E_. If *k* is too large, the computational cost in SOS analysis will be prohibitively high. We, therefore, aim to select a limited number of Galerkin modes from amongst the eigenfunctions (2.19) of the system (2.1) in such a way that the best stability bound can be obtained.

It is shown in appendix B that for *k*=4 with modes **e**_1,±1_,**e**_1,±2_ there is no polynomial Lyapunov function for the uncertain system (2.15)–(2.16) at *Re*>*Re*_E_. Note that in this case **f** is linear, while the proof of the existence of a polynomial Lyapunov functional given in [6] explicitly requires **f** to be quadratic, which of course can always be achieved by taking more Galerkin modes. For our particular flow, we therefore consider *k*≥6.

It is difficult to foresee the effect of mode selection on the system stability via the changes in **f**. It is also difficult to foresee the effect of mode selection on *p*(**a**,*q*^2^). However, minimizing *κ*_*s*_ is clearly beneficial. According to the definition (2.8), *κ*_*s*_ can be minimized simply by selecting the *k* modes of the finite basis to consist of the eigenfunctions with the largest eigenvalues λ_*n*,*m*_. Hence, we select the Galerkin modes by following such a criterion.

We define three sets of eigenfunctions {**e**_*n*,*m*_}:

$$\begin{array}{rl} K_{1} & =\{{\text{e}}_{1,-2},{\text{e}}_{1,-1},{\text{e}}_{1,0},{\text{e}}_{1,1},{\text{e}}_{2,-1},{\text{e}}_{2,0}\},\\ K_{2} & =K_{1}\cup \{{\text{e}}_{2,-2},{\text{e}}_{2,1}\}\\ \;\mathrm{and}\;K_{3} & =K_{2}\cup \{{\text{e}}_{1,-3},{\text{e}}_{1,2}\}. \end{array}$$

Table 1 presents the optimal selection of 6, 8 and 10 modes, respectively, for different *Re*∈(*Re*_E_,*Re*_E_+3.771), where *Re*_E_+3.771 is the value of *Re*_*L*_ at *Ω*=0.1. The mode selection result for *Re*≥*Re*_E_+3.771 can be derived similarly if needed.

**Table 1.** Selection of Galerkin modes for different *Re*∈(*Re*_E_,*Re*_E_+3.771).

*Re*	6 modes	8 modes	10 modes
(*Re*_E_,*Re*_E_+1.507)	*K*_1_	*K*_2_	*K*_3_
[*Re*_E_+1.507,*Re*_E_+2.828)	*K*_1_	{*K*_2_∖**e**_2,1_}∪**e**_1,−3_	*K*_3_
[*Re*_E_+2.828,*Re*_E_+3.611)	{*K*_1_∖**e**_1,1_}∪**e**_2,−2_	{*K*_2_∖**e**_2,1_}∪**e**_1,−3_	*K*_3_
[*Re*_E_+3.611,*Re*_E_+3.771)	{*K*_1_∖**e**_1,1_}∪**e**_2,−2_	{*K*_2_∖**e**_2,1_}∪**e**_1,−3_	{*K*_3_∖**e**_1,2_}∪**e**_2,−3_

### (b) Global stability of the flow

Proving that the flow is globally stable at a particular value of *Re* does not prove that it is globally stable for any other *Re*. However, for the flow in question (see §2b(ii)) there exists a global stability limit *Re*_G_ (equal to the linear stability limit *Re*_*L*_ for this flow) such that the flow is globally stable for all *Re*<*Re*_G_. Hence, any *Re* for which the flow is globally stable gives a lower bound for *Re*_G_. In particular, the SOS stability bound for the uncertain system *Re*_SOS_≤*Re*_G_.

The largest possible value of *Re*_SOS_ was obtained by trial end error. For a given *Re*, we try to find *V* satisfying the SOS constraints (2.14). If this is successful, we increase *Re* by *δRe* and repeat the trial. To do this, we assume some partially fixed structure of the candidate function *V*, that is the degree and the values of a part of the coefficients, and consider the remaining coefficients as the decision variables. We then tune the decision variables to satisfy (2.14), using the SOS package YALMIP [20], under which the decision variables were found using the semi-definite program (SDP) solver MOSEK [21]. Prior to solving the SDP, the SOS problem was pre-processed by the linear program solver GUROBI [22] in order to reduce and simplify the SOS program [23].

### (c) The best bound

The best result was achieved using the Galerkin mode set *K*_1_. For *K*_1_, (2.4) gives

$$\begin{array}{rl} f_{1} & =\left(-\frac{5}{Re}+\frac{\sqrt{5}}{5}\right)a_{1}-\frac{\sqrt{10}}{10\pi }a_{1}a_{6}+\frac{3\left(\sqrt{10}-8\right)}{80\pi }a_{2}a_{5}+\frac{3\left(\sqrt{10}+8\right)}{80\pi }a_{4}a_{5},\\ f_{2} & =\left(-\frac{2}{Re}+\frac{\sqrt{2}}{4}\right)a_{2}+\left(\frac{\sqrt{2}\Omega }{2}-\frac{\sqrt{2}}{4}\right)a_{4}-\frac{3\sqrt{10}}{40\pi }a_{1}a_{5}-\frac{1}{4\pi }a_{2}a_{6}+\frac{\sqrt{10}}{40\pi }a_{3}a_{5}+\frac{1}{4\pi }a_{4}a_{6},\\ f_{3} & =-\frac{1}{Re}a_{3}-\frac{5+\sqrt{10}}{40\pi }a_{2}a_{5}+\frac{5-\sqrt{10}}{40\pi }a_{4}a_{5},\\ f_{4} & =\left(\frac{\sqrt{2}}{4}-\frac{\sqrt{2}\Omega }{2}\right)a_{2}-\left(\frac{2}{Re}+\frac{\sqrt{2}}{4}\right)a_{4}-\frac{3\sqrt{10}}{40\pi }a_{1}a_{5}-\frac{1}{4\pi }a_{2}a_{6}+\frac{\sqrt{10}}{40\pi }a_{3}a_{5}+\frac{1}{4\pi }a_{4}a_{6},\\ f_{5} & =\left(\frac{\sqrt{5}}{10}-\frac{5}{Re}\right)a_{5}+\frac{3\left(8+\sqrt{10}\right)}{80\pi }a_{1}a_{2}+\frac{3\left(-8+\sqrt{10}\right)}{80\pi }a_{1}a_{4}+\frac{a_{2}a_{3}}{8\pi }-\frac{a_{3}a_{4}}{8\pi }\\ \;\mathrm{and}\;f_{6} & =-\frac{4}{Re}a_{6}+\frac{\sqrt{10}}{10\pi }a_{1}^{2}+\frac{1}{4\pi }a_{2}^{2}-\frac{1}{4\pi }a_{4}^{2}. \end{array}$$

For the flow in question it turns out that **Θ**_*a*_=0. Further, following the bound evaluation procedure introduced in §2a(ii), we have

3.1 $$\begin{array}{rl} |\Theta_{b}{|}^{2} & \leq \frac{q^{2}}{176800\pi^{2}}(291397a_{1}^{2}+(141083-340\sqrt{10})a_{2}^{2}+17680a_{3}^{2}+(141083+340\sqrt{10})a_{4}^{2}\\ & \;+269397a_{5}^{2}+85440a_{6}^{2}-194786a_{2}a_{4}), \end{array}$$

3.2 $$\begin{array}{rl} |\Theta_{c}{|}^{2} & \leq 1.401q^{4} \end{array}$$

3.3 $$\begin{array}{rl} \;\text{and}\;\kappa_{s} & =\left\{ \begin{array}{ll} 2\lambda_{2,-2}(Re)=-\frac{16}{Re}+\frac{\sqrt{2}}{2}, & Re\in (Re_{E},Re_{E}+2.282),\\ 2\lambda_{1,1}(Re)=-\frac{4}{Re}-\frac{\sqrt{2}}{2}, & Re\in [Re_{E}+2.282,Re_{E}+3.771). \end{array}\right. \end{array}$$

Unlike the simple calculation of *κ*_*s*_, estimating **Θ**_*b*_ and **Θ**_*c*_, while not very complicated, does involve certain amount of numerical calculations, such as optimization of finite-dimensional polynomials. The validity of the bounds (3.1) and (3.2) was partially verified by solving the Navier–Stokes equations (2.1) with boundary conditions (2.18) for five sets of initial conditions for the velocity. In each of the cases, calculations were performed with *Re*=2*Re*_L_,5*Re*_L_,10*Re*_L_ and *Ω*=0.1,0.25,0.5,0.75,0.9, giving 75 combinations in total. In each case, (3.1) and (3.2) hold true. The tightness of bound evaluation for each component of **Θ**_*b*_ and **Θ**_*c*_ was verified independently by maximizing them numerically over **u**_*s*_ under the constraint of ∥**u**_*s*_∥=1 for a set of fixed values **a**. All the tests gave satisfactory results.

The best stability bound was obtained with the following 4th-degree candidate function candidate:

3.4 $$V(\text{a},q^{2})=P(\text{a},{\text{c}}_{4})+P(\text{a},{\text{c}}_{2})q^{2}+\alpha q^{4},$$

where *α*≥0 and *P*(**a**,**c**_*i*_) is a general *i*th degree polynomial in **a**, with **c**_*i*_ denoting the coefficient vector. Noting that the constant term and the linear terms are obviously redundant in *V* (**a**,*q*^2^) owing to the first SOS constraint in (2.14), they are eliminated in advance. The Lyapunov functional with the structure of (3.4) can be adjusted by tuning the decision variables **c**_2_,**c**_4_ and *α*. Since $[{\text{c}}_{2},{\text{c}}_{4}^{T},\alpha ]\in R^{232}$ and $[\text{a},q,\text{z}]\in R^{14}$, there are 232 parametric variables and 14 independent variables for the SOS optimization. The parameters *ϵ*_*ij*_ required to construct the functions ℓ_*i*_ in (2.14) were fixed to

$$\epsilon_{1j}=1\times 10^{-5}\;\mathrm{and}\;\epsilon_{3j}=1.5\times 10^{-8}.$$

**Figure 1.**
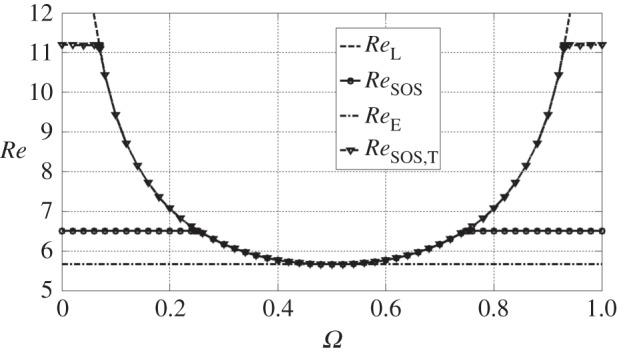
Stability results. *Re*_E_: energy stability limit of the flow (2.17)–(2.18). *Re*_L_: linear (and global) stability limit of the flow (2.17)–(2.18). *Re*_SOS_: SOS stability limit of the flow (2.17)–(2.18), obtained by solving the SOS problem for the uncertain system (2.15)–(2.16). *Re*_SOS,T_: SOS stability limit of the truncated system of ((2.15)–(2.16)), obtained by solving the corresponding SOS problem.

We then performed a trial-and-error procedure with *δRe*=0.01. Figure 1 shows the result. In the range *Ω*∈(0.2529,0.7471), *Re*_SOS_ coincides with *Re*_L_. Hence, in that range the method [6] produces the exact result. Outside this range the method gives only the lower bound for the true global stability limit *Re*_G_=*Re*_L_. This lower bound is still better than the bound *Re*_E_ obtained by the standard energy stability analysis.

The obtained bound is of the form

3.5 $$Re_{\mathrm{SOS}}(\Omega )=\min\{Re_{L}(\Omega ),Re_{E}+\Delta Re_{\mathrm{SOS}}\}=\min\{Re_{L}(\Omega ),Re_{E}+0.85\}.$$

The Lyapunov functionals obtained depend on *Ω*. For example, for *Ω*=0.1 it is

3.6 $$\begin{array}{rl} V(\text{a},q^{2}) & =7.9294{\left(|\text{a}{|}^{2}+2q^{2}\right)}^{2}-a_{5}(1.5894a_{1}a_{2}+3.1590a_{2}a_{3}-0.9151a_{1}a_{4}-0.0949a_{3}a_{4})\\ & \;-a_{6}(29.2233a_{2}^{2}-0.1480a_{1}a_{3}+4.8479a_{3}^{2}+2.6869a_{2}a_{4}+2.8354a_{4}^{2}+2.2428a_{5}^{2}+7.1675q^{2})\\ & \;+23.4772a_{1}^{2}+29.3767a_{2}^{2}+28.9780a_{3}^{2}+20.7968a_{4}^{2}+20.5949a_{5}^{2}+23.1982a_{6}^{2}+23.6155q^{2}\\ & \;-27.7365a_{2}a_{4}+0.0557a_{1}a_{3}. \end{array}$$

Some terms in (3.6) were grouped manually for brevity. In the original expanded form, (3.6) contains only 48 monomial terms in comparison to 232 possible monomials in (3.2). For different values of *Ω* the monomials themselves and their coefficients show some variation.

Note that the first term is proportional to the square of the total energy of the perturbation **u**. This was not imposed by us but was obtained as a result of the calculations. The likely form of the Lyapunov functional is discussed in [6].

These results demonstrate the feasibility of the method [6], thus achieving the primary goal of the present study.

### (d) Further observations

We remind that the stability of the flow at a given *Re* does not automatically imply stability at all smaller *Re*, even though such behaviour is typical. To simplify the exposition, we assume that our system does possess this typical behaviour. In fact, our results were obtained for a discrete, although reasonably dense, set of *Re* values.

The SOS stability limits obtained for the flow in question always turned out to be of the form $\min\{Re_{L}(\Omega ),Re_{E}+\Delta Re\},$ so that the constant Δ*Re* can be used as the measure of the quality of the bound. The stability bound *Re*_SOS,T_ of the truncated system (that is the system (2.15) with **Θ**=0) is also presented in figure 1. It is

3.7 $$Re_{\mathrm{SOS},T}=\min\{Re_{L},Re_{E}+5.52\}.$$

One can see that the uncertain term significantly reduces the SOS stability bound, namely, from Δ*Re*_SOS,T_=5.52 in (3.7) to Δ*Re*_SOS_=0.85 given by (3.5). Another immediate observation is that over a certain range of *Ω* the SOS bound for the stability margin of the truncated system is less than the true global stability margin of the full system, which coincides with *Re*_L_(*Ω*). These differences can be due to truncation, uncertainty and/or the use of SOS relaxation, in particular, the use of a not high-enough degree of the candidate Lyapunov function. In this section, we summarize the related observations.

#### (i) Stability limits of the full system and the truncated system

We observed that a better SOS stability limit of the truncated system does not necessarily imply a better SOS stability limit of the uncertain system.

For instance, we considered another set of six Galerkin modes: *K*_1_′={**e**_1,0_,**e**_1,±1_,**e**_2,0_,**e**_2,±1_}, which differs from the Galerkin set *K*_1_ in that the mode **e**_1,−2_ is replaced by **e**_2,1_. The SOS stability limit for the truncated *K*_1_′ system was found to be *Re*_SOS,T_=*Re*_L_(*Ω*), while it is $Re_{\mathrm{SOS},T}=\min\{Re_{L}(\Omega ),Re_{E}+5.52\}$ for *K*_1_. On the other hand, the SOS stability limits for the corresponding uncertain systems were found to be $Re_{\mathrm{SOS}}=\min\{Re_{L},Re_{E}+0.23\}$ for *K*_1_′ and $Re_{\mathrm{SOS}}=\min\{Re_{L},Re_{E}+0.85\}$ for *K*_1_.

This means that the selection of the Galerkin modes cannot be made on the basis of analysing the truncated system alone.

#### (ii) More Galerkin modes

Intuitively, it seems that increasing the number of Galerkin modes explicitly taken into account should improve the resulting SOS stability limit. The observations show a different picture.

For the Galerkin mode set *K*_2_, which includes the mode set *K*_1_ and two additional modes **e**_2,−2_ and **e**_2,1_, the uncertain fluid dynamical system is of the 8th order. In this case,

3.8 $$\begin{array}{rl} |\Theta_{b}{|}^{2} & \leq \frac{q^{2}}{884000\pi^{2}}((2959265+56940\sqrt{10})a_{1}^{2}+(1990190-45050\sqrt{10})a_{2}^{2}\\ & \;+127925a_{3}^{2}+(1990190+45050\sqrt{10})a_{4}^{2}+(2062505-61360\sqrt{10})a_{5}^{2}\\ & \;+541600a_{6}^{2}+(2657426+38064\sqrt{10})a_{7}^{2}+(2117755+61360\sqrt{10})a_{8}^{2}\\ & \;-2956730a_{5}a_{8}-3225580a_{2}a_{4}-332800a_{6}a_{7}-110500a_{1}a_{3}), \end{array}$$

3.9 $$\begin{array}{rl} |\Theta_{c}{|}^{2} & \leq 2.64q^{4} \end{array}$$

3.10 $$\begin{array}{rl} \;\text{and}\;\kappa_{s} & =\left\{ \begin{array}{ll} 2\lambda_{1,-3}(Re)=-\frac{20}{Re}+\frac{3\sqrt{10}}{10}, & Re\in (Re_{E},Re_{E}+1.507),\\ 2\lambda_{2,1}(Re)=-\frac{10}{Re}-\frac{\sqrt{5}}{5}, & Re\in [Re_{E}+1.507,Re_{E}+3.771). \end{array}\right. \end{array}$$

The expression for **f** is not shown here due to its large size. For a comparison with the 6-mode case, we consider the same 4th-degree Lyapunov function candidate (3.2). Owing to the increase in the number of the modes, a direct solution of the SOS optimization problem, (2.14) requires substantially greater computational effort than in the 6-mode case. For *K*_2_, there are 532 parametric variables and 18 independent variables in the SOS optimization. Solving the SOS problem (2.14) for *K*_2_ gives the SOS stability limit

3.11 $$Re_{\mathrm{SOS}}=\min\{Re_{L},Re_{E}+0.50\}$$

that is Δ*Re*_SOS,K_2__=0.50, which is less than Δ*Re*_SOS,K_1__=0.85.

For 10 Galerkin modes set *K*_3_ no feasible Lyapunov function was found for the SOS optimization problem (2.14), even after we decreased *Re* to *Re*_E_. In other words, Δ*Re*_SOS,K_3__=0.

To understand why increasing the number of Galerkin modes does not necessarily improve the SOS stability limit *Re*_SOS_, we revisit the stability condition for the uncertain system:

3.12 $$\frac{\partial V}{\partial \text{a}}\text{f}+\frac{\partial V}{\partial (q^{2})}\kappa_{s}q^{2}+\left|\frac{\partial V}{\partial \text{a}}-\frac{\partial V}{\partial (q^{2})}\text{a}\right|p^{\frac{1}{2}}(\text{a},q^{2})<0.$$

On the one hand, when more modes are taken into account, the negative *κ*_*s*_ becomes larger in magnitude, thus increasing the potential for (3.12) to be satisfied. However, using more Galerkin modes changes unfavourably the bounds of the uncertainties **Θ**_*b*_ and **Θ**_*c*_, thus increasing *p*(**a**,*q*^2^). As a result, the potential for (3.12) to be satisfied is reduced.

This means that there might be a finite optimum number of Galerkin modes to be included explicitly into the analysis by the method of Goulart & Chernyshenko [6].

#### (iii) Conservativeness analysis

Everywhere in this section we consider the *K*_1_ system, which gave the best SOS limits we obtained.

The global stability of the uncertain system implies the global stability of the Navier–Stokes system, but not the vice versa. The double-periodic rotating Couette flow (2.17)–(2.18) is globally stable for *Re*<*Re*_L_(*Ω*), while the best SOS stability limit we could obtain implies global stability for $Re<\min\{Re_{L}(\Omega ),Re_{E}+0.85\}$ only. This SOS stability limit can be conservative for two reasons. First, the limit obtained can be less than the actual stability limit for the uncertain system, for example because SOS approach gives only a sufficient condition for the non-negativity of a polynomial, or because the polynomial degree of the candidate Lyapunov function is not large enough. Second, it can be that the global stability limit for the uncertain system is smaller than the global stability limit for the full Navier–Stokes system. The second possibility turns out to be the case here for those values of *Ω* when *Re*_SOS_<*Re*_L_(*Ω*).

To demonstrate this, note that the obtained limits *Re*_SOS_ and *Re*_SOS,T_ are independent of *Ω* for sufficiently small or large *Ω*, as shown in figure 1. First, we consider *Re*_SOS,T._ It is easy to show analytically that the truncated system has five steady solutions:

$$O_{1}:(0,0,0,0,0,0),\;O_{2}^{\pm }:\left(\pm \frac{2\pi \sqrt{-50+2\sqrt{5}Re}}{Re},0,0,0,0,\frac{\sqrt{10}\pi (-25+\sqrt{5}Re)}{5Re}\right)$$

and

$$O_{3}^{\pm }:(0,a_{2}^{*},0,a_{4}^{*},0,a_{6}^{*}),$$

where

$$\begin{array}{rl} a_{2}^{*} & =\pm \frac{\sqrt{2}\pi \sqrt{Re^{2}(\Omega -\Omega^{2})-8}}{Re^{2}\Omega }(Re\Omega +2\sqrt{2}),\\ a_{4}^{*} & =\pm \frac{\sqrt{2}\pi \sqrt{Re^{2}(\Omega -\Omega^{2})-8}}{Re^{2}\Omega }(-Re\Omega +2\sqrt{2}),\\ \;\mathrm{and}\;a_{6}^{*} & =\frac{\sqrt{2}\pi (Re^{2}(\Omega -\Omega^{2})-8)}{Re^{2}\Omega }. \end{array}$$

The equilibria $O_{2}^{\pm }$ exist and are non-zero if $Re>5\sqrt{5}\approx Re_{E}+5.5235$. The equilibria $O_{3}^{\pm }$ exist and are non-zero if *Re*>*Re*_L_. Hence, the truncated system is not globally stable when $Re>\min\{Re_{L},5\sqrt{5}\}$, implying that $Re_{\mathrm{SOS},T}\leq \min\{Re_{L},5\sqrt{5}\}$. Combining the analytical stability result and the SOS optimization numerical result, one has

$$\min\{Re_{L},Re_{E}+5.52\}\leq Re_{\mathrm{SOS},T}\leq \min\{Re_{L},5\sqrt{5}\}\approx \min\{Re_{L},Re_{E}+5.5235\}.$$

It can be seen that the stability limit for the truncated system is attained by the SOS optimization analysis, in the sense that the error is less than *δRe*.

For the case of the uncertain system we could not obtain an analytic solution. Increasing the degree of the Lyapunov function candidate to six by taking

$$V(\text{a},q^{2})=P(\text{a},{\text{c}}_{6})+P(\text{a},{\text{c}}_{4})q^{2}+P(\text{a},{\text{c}}_{2})q^{4}+\alpha q^{6},$$

where **c**_2_,**c**_4_,**c**_6_ and *α* are decision variables, gave the same SOS stability limit as the 4th-degree Lyapunov function. Taking even higher degree polynomial led to so high computational costs that the calculations had to be abandoned.

Fortunately, we can demonstrate numerically that the SOS stability limit of the uncertain system obtained with the Lyapunov function of 4th degree is very close to the actual global stability limit of the uncertain system. For this reason, increasing the degree of Lyapunov function candidates is not helpful.

The idea is to evaluate the lower bound for such *Re* that there exist a steady non-zero solution of (2.15)–(2.16). This is similar to what we did for the truncated system, but we use numerical rather than analytic solutions. Naturally, the existence of steady non-zero solutions implies that the zero solution is not globally stable. Starting from any pre-specified Reynolds number for which there exists a non-zero equilibrium of the uncertain system, we decrease *Re* gradually. The smallest *Re* for which the uncertain system still has a non-zero equilibrium will be an upper bound of the global stability limit *Re*_*U*_ of the uncertain system.

Consider the following nonlinear optimization problem:

3.13 $$\begin{array}{rl} \min\; & Re\\ \text{s.t.}\; & \{(\text{a},\Theta ,\Gamma ,q)\;|\;F_{1},\ldots ,F_{4},|\text{a}{|}^{2}+q^{2}>0\}\neq \emptyset , \end{array}$$

where the constraints *F*_*i*_ are

$$\begin{array}{rl} & F_{1}:\text{f}(\text{a},Re)+\Theta =0,\\ & F_{2}:-\text{a}\Theta +\Gamma =0,\\ & F_{3}:\Gamma \leq \kappa_{s}(Re)q^{2}\\ \;\mathrm{and}\; & F_{4}:|\Theta {|}^{2}\leq p(\text{a},q^{2}). \end{array}$$

The constraints *F*_1_ and *F*_2_ ensure that $\dot{\text{a}}=\dot{\left(q^{2}\right)}=0$ in (2.15)–(2.16), and the constraints *F*_3_ and *F*_4_ are the bound for the uncertain terms in (2.15)–(2.16). In other words, we minimize *Re* subject to a constraint that there exist **a**,**Θ**,*Γ*,*q* satisfying a steady version of (2.15)–(2.16) and not coinciding with **a**=0,*q*=0 solution, the stability of which we are investigating. The variable *Γ* in the program can be eliminated by combining the constraints *F*_2_ and *F*_3_ as **aΘ**−*κ*_*s*_*q*^2^≤0. Since the constraints in (3.13) include equalities and inequalities and are highly nonlinear in *Re*, instead of solving (3.13) directly we consider the following optimization problem for a given *Re*:

3.14 $$\begin{array}{rl} \min\; & \phi (\omega_{1},\omega_{2},\epsilon )\\ \text{s.t.}\; & F_{1}:\text{f}(\text{a},Re)+\Theta =0, \end{array}$$

where $\phi (\omega_{1},\omega_{2},\epsilon )=\frac{1}{2}\sqrt{\varepsilon +{\left(\omega_{1}-\omega_{2}\right)}^{2}}+\frac{1}{2}(\omega_{1}+\omega_{2}),\omega_{1}={\text{a}}^{T}\Theta -\kappa_{s}(Re)q^{2},\omega_{2}=|\Theta {|}^{2}-p(\text{a},q^{2})$. In (3.14), the small parameter *ε*>0 is introduced to smooth the objective function. Note that

$$\phi (\omega_{1},\omega_{2},\epsilon )\geq \phi (\omega_{1},\omega_{2},0)=\frac{1}{2}|\omega_{1}-\omega_{2}|+\frac{1}{2}(\omega_{1}+\omega_{2})=\max(\omega_{1},\omega_{2}).$$

Hence, negative *ϕ*(*ω*_1_,*ω*_2_,*ϵ*) implies *ω*_1_<0,*ω*_2_<0. As such, all the constraints in (3.13) would be satisfied if *ϕ*(*ω*_1_,*ω*_2_,*ϵ*)<0 in (3.14). Now, the lower bound for *Re* that leads to the non-global stability of system (2.15)–(2.16) can be obtained by decreasing *Re* and solving (3.14) repeatedly. This procedure is stopped once the minimum of *ϕ*(*ω*_1_,*ω*_2_,*ϵ*) is no longer negative.

Let *ϵ*=0.1 in (3.14). Tables 2 and 3 show the optimization results for *Ω*=0 and *Ω*=0.1 when *Re* decreases from *Re*_E_+1.00 to *Re*_E_+0.85. The initial searching point in each trial is always set as the stopping point in the previous trial. The sequential quadratic programming method [24] associated with the function NLPSolve in MAPLE optimization toolbox is used to solve (3.14). From the tables, we can see that all the constraints in (3.13) can be satisfied when *Re*≥*Re*_E_+0.86 in the sense that the residual error |**f**+**Θ**|^2^ is negligible. This implies that the global stability limit for the uncertain system is less than *Re*_E_+0.86. Recalling that *Re*_SOS_=*Re*_E_+0.85 for *Ω*=0 and *Ω*=0.1, we conclude that at least for these values of *Ω* the actual global stability limit for the uncertain system is between *Re*_SOS_ and *Re*_SOS_+0.01. The verification for other values of *Ω* can be conducted similarly.

**Table 2.** Solution of the optimization problem (3.14) for different *Re* and *Ω*=0.1.

*Re*−*Re*_E_	*ω*_1_	*ω*_2_	|**f**+**Θ**|^2^	(**a**,*q*)
1.00	−290.07	−332.17	0.93×10^−12^	(−1.32, 190.85,0.80,−5.87,0.04,0.80,30.91)
0.95	−462.26	−548.04	0.23×10^−10^	(0.47,297.91,0.13,−6.60,1.21,0.83,48.42)
0.90	−450.69	−542.75	0.24×10^−10^	(0.48,429.18,0.43,−12.41,0.03,0.45,69.56)
0.88	−283.40	−388.67	0.36×10^−14^	(7.51,492.09,1.03,−17.94,−1.03,1.05,79.54)
0.86	−44.74	−151.69	0.15×10^−9^	(−0.0007,497.4, 0.022,−14.32,0.088,0.72,80.62)
0.85	89.36	−13.42	0.40×10^−14^	(−0.09,478.46,−0.066,−13.80,−0.002,0.60,77.55)

**Table 3.** Solution of the optimization problem (3.14) for different *Re* and *Ω*=0.

*Re*−*Re*_E_	*ω*_1_	*ω*_2_	|**f**+**Θ**|^2^	(**a**,*q*)
1.00	−1521.06	−1615.22	0.23×10^−10^	(3.10,441.36,−4.05,−19.06,−1.33,−1.71,71.11)
0.95	−991.75	−1086.27	0.25×10^−13^	(−1.91,437.43,2.73,−11.94,3.98,1.33,70.95)
0.90	−613.87	−695.01	0.23×10^−9^	(1.01,501.28,0.40,−14.29,−0.58,−0.08,81.27)
0.88	−491.01	−870.92	0.60×10^−9^	(0.23,610.89,0.51,−16.50,0.23,0.55,99.09)
0.86	−84.21	−230.21	0.22×10^−9^	(−0.001,682.29, 0.0003,−19.66,0.06,0.78,110.59)
0.85	177.66	7.68	0.22×10^−9^	(−0.74,673.95,−0.34,−19.44,−0.20,1.03,109.24)

Overall, the analysis of this section shows that for the flow in question further improvement of the SOS stability limit can be achieved only by improving the uncertainty bounds, since increasing the number of Galerkin modes explicitly taken into account gives no improvement, while the SOS stability limit for the uncertain system is already tight.

## 4. Discussion and conclusion

The uncertainty bounds obtained by the methods proposed in [6] and appendix A are tight for each of the components of **Θ**_*b*_ and **Θ**_*c*_. However, they are attained at different **a** and **u**_*s*_. Hence, the bound on |**Θ**| used to obtain the stability limit is not tight. This provides a potential for improving the results.

For all practical purposes, the question of a global stability of a particular flow can often be answered by physical experiment or numerical calculation, at least up to the existence of unstable stationary points or orbits. Theoretical studies of global stability, however, can provide deeper insight into various aspects of the flow and of the methods used. For example, one could hope to gain such insight from the particular form of the Lyapunov functional. It appears, however, that the method [6] generates Lyapunov functionals of a rather complicated form, which is difficult to interpret. In addition, the obtained Lyapunov functional depends on computational parameters such as the number of the Galerkin modes taken explicitly into account and the degree and the form of the polynomial representing the candidate Lyapunov function.

Reduction of the Navier–Stokes equation to an uncertain system, which forms the basis of the method of [6], turns out to be useful for a number of problems, which might be even of larger interest than the global stability problem. For example, it might prove useful in studies of bounds for long-time averages of the characteristics of turbulent flows and other infinite-dimensional systems with complicated behaviour, and in designing control for such systems [14]. For such studies, the observations of the properties of the method of [6] made in the present work might constitute even a greater interest than the central result of this paper.

It remains to give a summary of the obtained results and observations.

A new expression for one of the uncertainty bounds required by the method of [6] was obtained (see appendix A). It allows a considerable reduction of the computational cost as compared to the approach proposed in [6].

A systematic approach to selecting the particular Galerkin modes for the uncertain system was proposed.

It was shown that the selection of Galerkin modes leading to a better stability result for the truncated Galerkin system does not necessarily lead to a better stability result for the uncertain system.

It was also shown that increasing the dimension of the uncertain system does not necessarily improve the stability bound obtained for the full system, and that increasing the degree of the candidate polynomial Lyapunov function also does not necessarily improve the bounds. This suggests that further progress in this problem is more likely to be achieved by improving the bounds in the uncertain system than by improving SOS optimization.

For the particular version of the double-periodic rotating Couette flow we considered, we demonstrated that

(i) for *Ω*∈(0.2529,0.7471), the SOS stability limit coincides with the actual global stability limit.
(ii) for *Ω*∈(0,0.2529]∪[0.7491,1), the SOS stability limit *Re*_SOS_≥*Re*_E_+0.85,

where *Re*_E_ is the energy stability limit. This demonstrates the feasibility of the method proposed in [6].

To the best of our knowledge, this is the first case in which a systematic method applicable in principle to any fluid flow was used successfully to prove global stability of a fluid flow for the value of the Reynolds number greater than that which could be achieved with the energy stability approach.

## Footnotes

1 *V* (**a**,*q*^2^) is a functional of **u** because *a*_*i*_=〈**u**,**u**_*i*_〉 and $q=∥\text{u}-\sum_{i=1}^{k}\langle \text{u},{\text{u}}_{i}\rangle {\text{u}}_{i}∥/\sqrt{2}$ are functionals of **u**.

2 Note a misprint in equation (28) in [6]: a missing factor of 2.

## Appendix A. Bound evaluation for **Θ**_**c**_(**u**_*s*_)

Note that **u**_*s*_⋅∇**u**_*i*_⋅**u**_*s*_=**u**_*s*_⋅*D*_*i*_(**x**)⋅**u**_*s*_, where *D*_*i*_(**x**)=(∇**u**_*i*_+∇^*T*^ **u**_*i*_)/2 are the rate of strain tensors of the Galerkin basis vector fields. Hence, for *i*=1,…,*k*,

A 1 $$\Theta_{\text{c}i}({\text{u}}_{s})=\langle {\text{u}}_{s},{\text{u}}_{s}\cdot \nabla {\text{u}}_{i}\rangle =\int_{V}{\text{u}}_{s}\cdot D_{i}\cdot {\text{u}}_{s}\;dV=\int_{V}\frac{{\text{u}}_{s}^{T}D_{i}{\text{u}}_{s}}{|{\text{u}}_{s}{|}^{2}}|{\text{u}}_{s}{|}^{2}\;dV.$$

Then, we can see that

A 2 $$|\Theta_{\text{c}i}({\text{u}}_{s})|\leq \int_{V}|\frac{{\text{u}}_{s}^{T}D_{i}{\text{u}}_{s}}{|{\text{u}}_{s}{|}^{2}}∥{\text{u}}_{s}{|}^{2}\;dV\leq \int_{V}{\bar{D}}_{i}(\text{x})|{\text{u}}_{s}{|}^{2}\;dV\leq 2|{\bar{D}}_{i}(\text{x}){|}_{\infty }q^{2},$$

where $|\cdot {|}_{\infty }$ is the supremum norm and ${\bar{D}}_{i}:V\to R$ is defined point-wise for x∈V as

$${\bar{D}}_{i}(\text{x})=\underset{\text{w}\in R^{3}}{\sup}\left|\frac{{\text{w}}^{T}D_{i}(\text{x})\text{w}}{|\text{w}{|}^{2}}\right|.$$

Since what is inside the absolute value is the well-known Rayleigh quotient whose maximum and minimum are the largest and smallest eigenvalues of *D*_*i*_(**x**), it is immediate that ${\bar{D}}_{i}(\text{x})$ is in fact the spectral radius of *D*_*i*_(**x**), which as usual is denoted by *ρ*(*D*_*i*_(**x**)). As such, $|\rho (D_{i}(\text{x})){|}_{\infty }$ is the global maximum of the spectral radii constrained to x∈V, and

A 3 $$|\Theta_{\text{c}i}({\text{u}}_{s})|\leq 2|\rho (D_{i}(\text{x})){|}_{\infty }q^{2}.$$

The bound (A3) can be shown to be tight by constructing a function **u**_*s*_ with compact support similar to a delta function centred around the point **x**_*m*_, where *ρ*(*D*_*i*_(**x**)) attains its global maximum. The idea here is similar to the one used in [25], while the difference is that **u**_*s*_ is a vector and not a scalar, and that it has to be solenoidal, and orthogonal to all the basis field functions **u**_*i*_,*i*=1,…,*k*.

Now, by (A3), this yields the tight bound

$$|\Theta_{\text{c}}({\text{u}}_{s}){|}^{2}=\sum_{i=1}^{k}\Theta_{ci}^{2}({\text{u}}_{s})\leq 4\sum_{i=1}^{k}|\rho (D_{i}(\text{x})){|}_{\infty }^{2}q^{4}.$$

## Appendix B. Non-existence of polynomial Lyapunov functions

An example is given to illustrate that an excessively low-order uncertain system may not be suitable in the sense that there does not exist a polynomial Lyapunov function that guarantees its stability when *Re*>*Re*_E_.

In this case, the uncertain system (2.15)–(2.16) involves only four modes. More precisely, let **u**_1_=**e**_1,1_,**u**_2_=**e**_1,−1_,**u**_3_=**e**_1,2_ and **u**_4_=**e**_1,−2_. It is easy to calculate that

$$\begin{array}{rl} f_{1} & =-\left(\frac{2}{Re}+\frac{\sqrt{2}}{4}\right)a_{1}+\frac{\sqrt{2}}{4}(1-2\Omega )a_{2},\\ f_{2} & =-\frac{\sqrt{2}}{4}(1-2\Omega )a_{1}+\left(\frac{\sqrt{2}}{4}-\frac{2}{Re}\right)a_{2},\\ f_{3} & =-\left(\frac{5}{Re}+\frac{\sqrt{5}}{5}\right)a_{3}+\frac{\sqrt{5}}{5}(1-2\Omega )a_{4}\\ \;\mathrm{and}\;f_{4} & =-\frac{\sqrt{5}}{5}(1-2\Omega )a_{3}+\left(\frac{\sqrt{5}}{5}-\frac{5}{Re}\right)a_{4}. \end{array}$$

The bounds for the uncertain terms **Θ**(**u**_*s*_,**a**) and *Γ*(**u**_*s*_) are, respectively,

B 1 $$\begin{array}{rl} p(\text{a},q^{2}) & =\frac{q^{2}}{1300\pi^{2}}(1091a_{1}^{2}-1571a_{1}a_{2}+1091a_{2}^{2}+875a_{3}^{2}-1140a_{3}a_{4}\\ & \;+875a_{4}^{2}+10140q^{2}+1300\sqrt{3}q^{2})\\ \;\mathrm{and}\;\kappa_{s} & =2\max_{m\in Z\setminus \{-1,-2\}}\lambda_{1,m}(Re)<0. \end{array}\}$$

Recall that for a positive definite Lyapunov candidate function of the form *V* =*V* (**a**,*q*^2^) satisfying ∂*V* /∂*q*^2^≥0, a sufficient condition for stability is (2.11), i.e.

B 2 $$\frac{\partial V}{\partial \text{a}}\text{f}+\frac{\partial V}{\partial (q^{2})}\cdot \kappa_{s}q^{2}<-\left|\frac{\partial V}{\partial \text{a}}-\frac{\partial V}{\partial (q^{2})}\text{a}\right|p^{1/2}(\text{a},q).$$

For *Re*<*Re*_E_, the total energy *E*_*t*_=|**a**|^2^/2+*q*^2^ is the Lyapunov function as |∂*E*_*t*_/∂**a**−∂*E*_*t*_/∂(*q*^2^)**a**|=0 and (∂*E*_*t*_/∂**a**)**f**+∂*E*_*t*_/∂(*q*^2^)⋅*κ*_*s*_*q*^2^<0. However, this is not the case for *Re*>*Re*_E_, namely, there does not exist a polynomial Lyapunov function for the uncertain system (2.15)–(2.16), if only the modes **e**_1,±1_,**e**_1,±2_ are considered.

We first prove that the total energy *E*_*t*_ is not a Lyapunov function. Note that $(\partial E_{t}/\partial \text{a})\text{f}+\partial E_{t}/\partial (q^{2})\cdot \kappa_{s}q^{2}=\lambda_{1,1}a_{1}^{2}+\lambda_{1,-1}a_{2}^{2}+\lambda_{1,2}a_{3}^{2}+\lambda_{1,-2}a_{4}^{2}+\kappa_{s}q^{2}$, where $\lambda_{1,1}<0,\lambda_{1,-1}=2/Re_{E}-2/Re,\lambda_{1,2}<0,\lambda_{1,-2}=\sqrt{5}/5-5/Re$. When *Re* increases and exceeds *Re*_E_, λ_1,−1_ will be the first to become positive, implying that *E*_*t*_ is not a Lyapunov function.

Moreover, as |a|→∞ and q→∞, the leading term of *V* cannot be proportional to *E*_*t*_. Indeed, let $V=E_{t}^{n}+V_{1}$, such that $V_{1}\ll E_{t}^{n}$ as |a|→∞. Then, since **f**(**a**)=*O*(|**a**|), $\left(\partial V_{1}/\partial \text{a})\text{f}∼V_{1}\ll (\partial E_{t}^{n}/\partial \text{a})\text{f}=nE_{t}^{n-1}(\lambda_{1,1}a_{1}^{2}+\lambda_{1,-1}a_{2}^{2}+\lambda_{1,2}a_{3}^{2}+\lambda_{1,-2}a_{4}^{2}\right)$. Hence, for large enough *a*_2_ and *a*_1_=*a*_3_=*a*_4_=*q*=0, (∂*V* /∂**a**)**f**>0, so that *V* is not a Lyapunov function.

On the other hand, in view of (B1), *p*(**a**,*q*^2^)=*O*(*q*^2^(|**a**|^2^+*q*^2^)). If the main term of *V* is not a function of *E*_*t*_, then the order of the right-hand side of (B2) is greater than the order of its left-hand side, and the inequality (B2) cannot hold true, so that *V* cannot be a Lyapunov function in this case either.

Overall, when *Re*>*Re*_E_, there does not exist a polynomial Lyapunov function for the uncertain system (2.15)–(2.16), if only the modes **e**_1,±1_,**e**_1,±2_ are considered.
